# The Nicotinic Acetylcholine Receptors of the Parasitic Nematode *Ascaris suum*: Formation of Two Distinct Drug Targets by Varying the Relative Expression Levels of Two Subunits

**DOI:** 10.1371/journal.ppat.1000517

**Published:** 2009-07-17

**Authors:** Sally M. Williamson, Alan P. Robertson, Laurence Brown, Tracey Williams, Debra J. Woods, Richard J. Martin, David B. Sattelle, Adrian J. Wolstenholme

**Affiliations:** 1 Department of Biology & Biochemistry, University of Bath, Bath, United Kingdom; 2 Department of Biomedical Sciences, Iowa State University, Ames, Iowa, United States of America; 3 MRC Functional Genomics Unit, Oxford, United Kingdom; 4 Veterinary Medicine Research & Development, Pfizer Animal Health, Kalamazoo, Michigan, United States of America; Queen's University Belfast, United Kingdom

## Abstract

Parasitic nematodes are of medical and veterinary importance, adversely affecting human health and animal welfare. *Ascaris suum* is a gastrointestinal parasite of pigs; in addition to its veterinary significance it is a good model of the human parasite *Ascaris lumbricoides*, estimated to infect ∼1.4 billion people globally. Anthelmintic drugs are essential to control nematode parasites, and nicotinic acetylcholine receptors (nAChRs) on nerve and muscle are the targets of cholinergic anthelmintics such as levamisole and pyrantel. Previous genetic analyses of nematode nAChRs have been confined to *Caenorhabditis elegans*, which is phylogenetically distinct from *Ascaris spp.* and many other important parasites. Here we report the cloning and expression of two nAChR subunit cDNAs from *A. suum*. The subunits are very similar in sequence to *C. elegans* UNC-29 and UNC-38, are expressed on muscle cells and can be expressed robustly in *Xenopus* oocytes to form acetylcholine-, nicotine-, levamisole- and pyrantel-sensitive channels. We also demonstrate that changing the stoichiometry of the receptor by injecting different ratios of the subunit cRNAs can reproduce two of the three pharmacological subtypes of nAChR present in *A. suum* muscle cells. When the ratio was 5∶1 (*Asu-unc-38∶Asu-unc-29*), nicotine was a full agonist and levamisole was a partial agonist, and oocytes responded to oxantel, but not pyrantel. At the reverse ratio (1∶5 *Asu-unc-38∶Asu-unc-29*), levamisole was a full agonist and nicotine was a partial agonist, and the oocytes responded to pyrantel, but not oxantel. These results represent the first *in vitro* expression of any parasitic nicotinic receptor and show that their properties are substantially different from those of *C. elegans*. The results also show that changing the expression level of a single receptor subunit dramatically altered the efficacy of some anthelmintic drugs. *In vitro* expression of these subunits may permit the development of parasite-specific screens for future anthelmintics.

## Author Summary

Ascarid nematodes are major pathogens of humans and livestock. The major method of control is by the use of anthelmintic drugs, many of which target the nervous system. Drugs such as levamisole, pyrantel and oxantel target the nicotinic acetylcholine receptors present on muscle. Nematodes have several such receptors, and until now these have been best understood in the model species *Caenorhabditis elegans*. We have started to characterise the nicotinic receptors of *Ascaris suum*, and find that the genetics and pharmacology of the *A. suum* receptors differ from *C. elegans*. In both species, nicotine and levamisole preferentially activate different forms of the nicotinic receptor, the N- and L-type, respectively. In *C. elegans*, the L-type receptor is made up of five subunits, whereas the N-type is a homomer of a sixth subunit. We can recapitulate many of the properties of the *A. suum* N- and L-type receptors, including their sensitivity to two other important anthelmintics, pyrantel and oxantel, by expressing just two subunits at varying ratios. This has implications for the use of drug combinations and for cross-resistance between nicotinic anthelmintics. It may start to give an explanation for the varying effectiveness of nicotinic drugs against different parasites.

## Introduction

Nematodes of the genus *Ascaris* are large (∼30 cm) gastrointestinal parasites of swine (*Ascaris suum*) and humans (*Ascaris lumbricoides*). *Ascaris lumbricoides* infects ∼1.4 billion people globally, and is particularly prevalent in conditions of poor sanitation and poverty [1],[2]. In addition to directly causing morbidity (such as malnutrition) and mortality (via obstruction of the gut or bile duct), *A. lumbricoides* infection also exacerbates other diseases prevalent in impoverished communities such as malaria, tuberculosis and AIDS [3],[4]. *Ascaris suum* is a major parasite of pigs, causing serious economic losses for farmers [5]. It could also be considered as a good model of the human parasite and in some cases may infect humans directly as a zoonosis [6],[7].

At present, control of helminth infections in both animals and humans relies on administration of anthelmintic chemotherapeutic agents. The nematode nicotinic acetylcholine receptors (nAChRs) on muscle are the target of the cholinergic anthelmintics levamisole, pyrantel and oxantel; such compounds cause these ligand-gated ion channels to open, leading to prolonged muscle contraction and spastic paralysis of the parasite [8]. The importance of nematode nAChR as drug targets has been underlined by the recent announcement of a new class of cholinergic anthelmintics, the amino-acetonitrile derivatives, though these do not seem to act on muscle nicotinic receptors [9]. The relative ease with which the large muscle cells of *A. suum* can be manipulated has permitted electrophysiological characterisation of the native nAChRs. This has shown that 3 distinct pharmacological nAChR subtypes are present on *Ascaris* muscle cells, with different agonist and antagonist sensitivities: an L-subtype most sensitive to the agonists levamisole and pyrantel, an N-subtype most sensitive to nicotine, oxantel and methyridine, and a B-subtype most sensitive to bephenium [10]–[12]. Nicotine- and levamisole-sensitive nAChR have also been identified in the model nematode *C. elegans*, where the genes *unc-38*, *unc-29*, *unc-63*, *lev-1* and *lev-8* encode nAChR subunits involved in levamisole sensitivity [13]. Heterologous expression of *unc-38*, *unc-29* and *lev-1* in the *Xenopus* oocyte system produces a functional levamisole-sensitive nAChR, but the currents have a small amplitude suggesting the need for additional components [14]–[16]. Recently Boulin et al [17] have reported that eight gene products, the five receptor subunits and three ancillary proteins (RIC-3, UNC-50 and UNC-74) are required to reconstruct the L-type receptor from *C. elegans*. This receptor is completely insensitive to nicotine, but nicotine-sensitive receptors are present in *C. elegans* and are formed by a pentamer of ACR-16 subunits, which express robustly in *Xenopus* oocytes [18]. However, *Ascaris spp.* and several other medically important parasites (for instance *Brugia malayi* and *Onchocerca volvulus*, which cause lymphatic filariasis and river blindness) belong to a phylogenetic group (clade III) which is only distantly related to *C. elegans*, which is a member of clade V [19]. We have previously reported that many nAChR subunit genes found in *C. elegans* are absent from the *B. malayi* genome [20]. Of particular relevance is the absence of sequences similar to the nAChR subunit genes *lev-1* and *lev-8*, which encode subunits of the *C. elegans* L-type receptor, and a greatly reduced number of genes encoding subunits similar to ACR-16, which makes up the N-type receptor [21]. This analysis suggests that the subunit composition of the L- and N-type nicotinic receptors may be quite different in the pathogenic clade III nematodes from that found in *C. elegans*. However, there have been very few molecular studies on parasitic nematode nicotinic receptors, and their subunit composition has not been determined to date.

We have therefore been following up this bioinformatic analysis with molecular studies, with the intention of recapitulating the L- and N-subtypes of the *A. suum* nAChR *in vitro*. Here we report the identification of *A. suum* orthologues of *unc-38* and *unc-29*, and confirm, using antibody labelling, their presence and co-localisation in *A. suum* muscle cells. When expressed in *Xenopus* oocytes they form functional levamisole-sensitive nAChR. By altering the ratio at which the two cRNAs encoding these subunits are injected into the oocytes, receptors with different pharmacological profiles can be produced that respond to different nicotinic anthelmintics. The pharmacology of these receptors resembles the L- and N-subtypes present in native *A. suum* muscle cells.

## Results

### Cloning and antibody labelling

**Figure 1:**
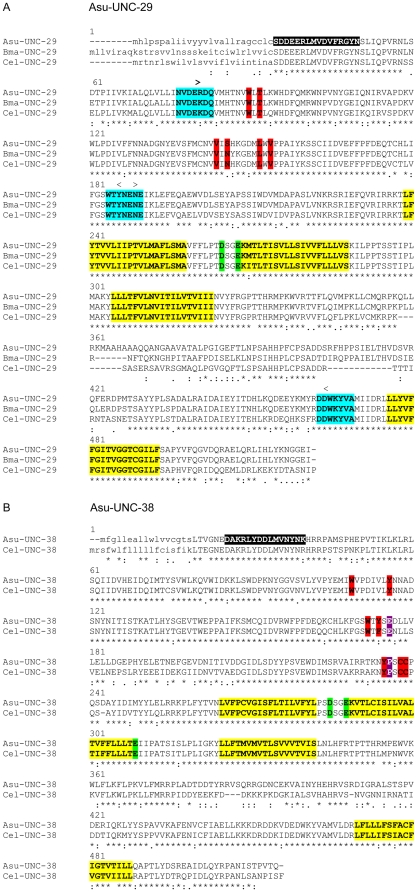
The translated sequence of Asu-UNC-29 and Asu-UNC-38 aligned with the *C. elegans* and *B. malayi* equivalents (UNC-29 only). In panel A), the conserved regions used in the design of the degenerate oligonucleotides are highlighted in blue. For both panels, the predicted signal peptide is in lower case, the transmembrane regions are highlighted in yellow, the ligand binding residues [39] in red, the glutamic acid and aspartic acid residues conferring cation specificity to the ion channel [40] are in highlighted in green. The N-terminal subunit-specific peptides used to raise antisera are highlighted in black. In panel B), the amino-acid residues identified by Rayes et al [24] as essential for levamisole activation are highlighted in purple.

In order to obtain cDNAs encoding nicotinic receptor subunits from *A. suum*, we first aligned the amino-acid sequence of the *C. elegans* UNC-38 and UNC-29 subunits with their predicted orthologues from *B. malayi*. A partial cDNA sequence from *A. suum* similar to *C. elegans unc-38* was found in the database (Accession number AJ011382) and used to design specific primers to extend the sequence. Degenerate primers based on well conserved regions of amino-acid sequence were used to amplify a partial cDNA clone of the *unc-29*-like gene from *A. suum* (Figure 1). 5′ and 3′ RACE procedures [22] were then used to obtain the remainder of the sequences. Full-length cDNAs encoding the *A. suum* orthologues of *unc-38* and *unc-29* were then amplified using primers specific for the 5′ and 3′ termini, cloned, sequenced and deposited in GenBank with the accession numbers EU053155 and EU006073. The predicted sequences of the subunits, designated Asu-UNC-29 and Asu-UNC-38 (using the nomenclature proposed in http://www.wormbase.org/wiki/index.php/UserGuide:Nomenclature_nematode) are shown in Figure 1, together with an alignment with the *C. elegans* and predicted *B. malayi* subunits. The residues within the predicted loops that make up the ligand-binding site [23], predicted signal peptide and transmembrane domains, and the residues predicted to confer cation specificity on the ion channel are highlighted. The sequences predict that Asu-UNC-38 is an α subunit, since it contains the typical Y-X(X)-CC ACh binding motif (residues 185–190) in loop C of the ligand-binding site, whereas Asu-UNC-29, lacking this motif, has all the characteristics of a non-α subunit, with complementary binding residues. Asu-UNC-38, like its *C. elegans* equivalent, also possesses the amino-acid residues reported to be necessary for activation by levamisole at positions 153 (glutamate) and 191 (proline) of the mature polypeptide [24]. The designation of these subunits as the *A. suum* equivalents of UNC-29 and UNC-38 is based on the results of BLASTP searches and pair-wise sequence comparisons with the *C. elegans* nicotinic receptor subunits (Table 1).

**Table 1:** Comparison of the amino-acid sequences of *A. suum* nicotinic receptor subunits with those of *C. elegans*.

*C. elegans* polypeptides in order of identity to *Ascaris suum* UNC-38	Percentage identity	*C. elegans* polypeptides in order of identity to *Ascaris suum* UNC-29	Percentage identity
UNC-38	80	UNC-29	77
UNC-63	53	LEV-1	62
ACR-12	38	ACR-3	55
ACR-8	40	ACR-2	49
ACR-6	36	UNC-63	37

The best hits from a BLASTP search are shown.

**Figure 2:**
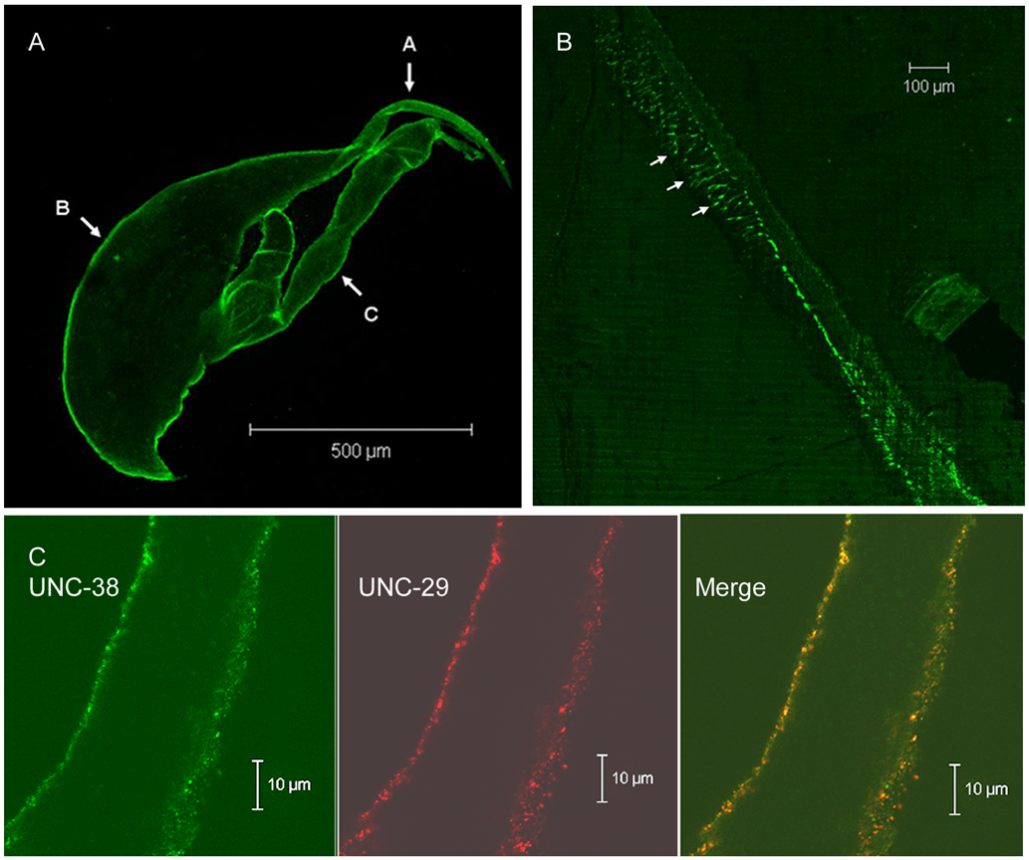
Immunolocalisation of nAChR subunits in isolated *A. suum* muscle cells. A) Confocal microscope images showing indirect immunofluorescent labelling of *Ascaris suum* muscle with primary antibody against Asu-UNC-38 and FITC conjugated secondary antibody. A whole muscle cell is shown, with Asu-UNC-38 localised to the cell membrane over the entire cell surface. Arrow A indicates the muscle cell arm, B the bag region of the cell, and C the contractile region. B) Asu-UNC-38 localised to the muscle arms where contact with the nerve cord is made to form the neuromuscular junction (indicated by arrows). No labelling of the nerve cord itself was observed. C) Co-localisation of Asu-UNC-29 and Asu-UNC-38 on the muscle cell arm (region A in panel A).

If Asu-UNC-29 and Asu-UNC-38 are components of the *A. suum* L- or N-type nicotinic receptors, they should be expressed on muscle cells. We therefore raised specific anti-subunit antibodies, using synthetic peptides from the N-terminal region of the predicted mature subunits as immunogens (Figure 1). The resultant antibodies were purified by affinity chromatography and used to perform indirect immunofluorescence on whole muscle cells obtained by collagenase treatment of *A. suum*. The anatomy of *Ascaris spp.* muscle cells and neuromuscular junctions is somewhat different from that of mammals [25], in that the muscle cells send out processes, muscle arms, which make contact with the neurons within the nerve cord; the neuromuscular junctions are thus at the ends of the muscle arms. Figure 2A shows that the anti-Asu-UNC-38 antibody labelled the whole surface of isolated muscle cells, including the muscle arms (indicated by arrow A), the bag region, which is used in the preparation of the patches used in the single channel recordings [10]–[12], and spindle which is the contractile region. The same antibody also labelled portions of the nerve cord (Figure 2B) and portions of the muscle arms that had remained attached to the cord during sample preparation. Antibodies against both subunits labelled the muscle cell membrane in the muscle arm region which leads from the muscle cell to the nerve to form the neuromuscular junction (Figure 2C). The antibody labelling also showed that the expression of the two subunits overlapped, suggesting they may be components of the same receptor. All of these results are consistent with these two subunits being components of a muscle receptor, which is expressed extra-synaptically and synaptically at the neuromuscular junction.

### Co-expression of Asu-UNC-38 and Asu-UNC-29 produces functional heteromeric nAChRs

**Figure 3:**
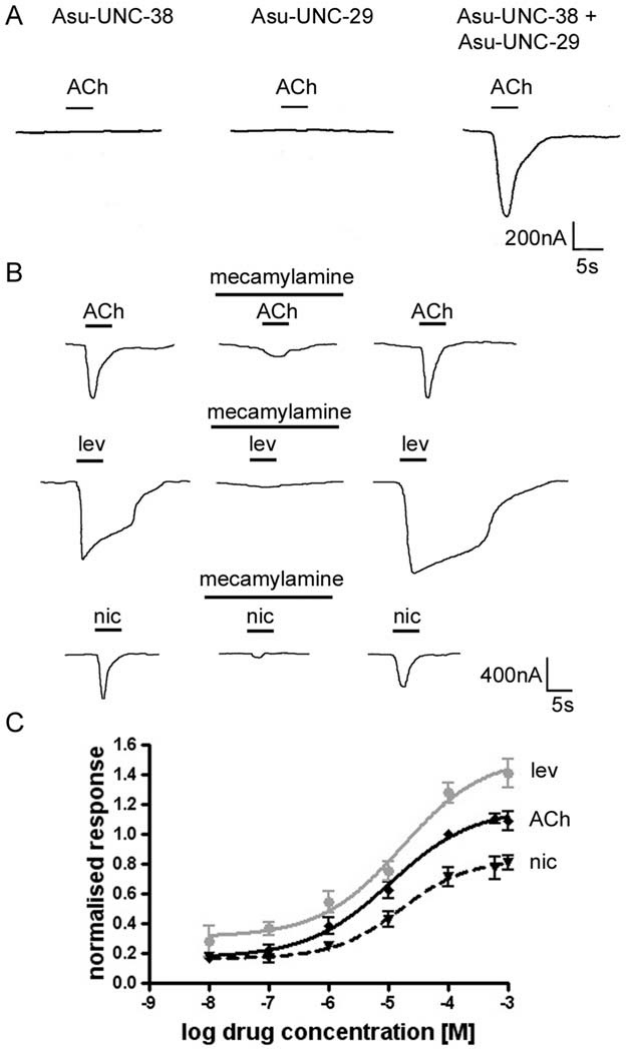
Two-electrode voltage clamp electrophysiological experiments on *Xenopus* oocytes. A) Oocytes injected with *Asu- unc-38* or *Asu-unc-29* cRNAs alone produced no functional receptors, whereas oocytes injected with both subunit cRNAs in an equimolar ratio gave robust responses to application of 100 µM acetylcholine (ACh). B) Oocytes injected with an equimolar ratio of *Asu-unc-38* and *Asu-unc-29* cRNA responded to 100 µM acetylcholine (ACh), 100 µM levamisole (lev) and 100 µM nicotine (nic). These responses could all be reversibly blocked by application of 10 µM mecamylamine. C) Dose-response relationships for the agonists levamisole (lev, grey circles), acetylcholine (ACh, black diamonds) and nicotine (nic, black triangles) from oocytes injected with an equimolar ratio of *Asu-unc-38* and *Asu-unc-29*. Responses are all normalized to the response to 100 µM acetylcholine. N = 3 (where N is batches of oocytes) and n = 6 (where n is the number of individual oocytes) minimum for each data point.

Since the two subunits seemed to co-localise on muscle cells and may thus be predicted to co-assemble in the same receptors, we injected cRNAs encoding the two polypeptides into *Xenopus* oocytes. Two-electrode voltage clamp experiments showed that Asu-UNC-38 and Asu-UNC-29 formed a heteromeric receptor when expressed in these cells. The results are shown in Figure 3: injection of neither cRNA produced functional receptors when injected into *Xenopus* oocytes alone, but when equal amounts of cRNAs encoding both subunits were injected, functional nAChRs were produced which gave a robust (∼800 nA) response to 100 µM acetylcholine (Ach). The receptors also responded to 100 µM levamisole and 100 µM nicotine, and all of these responses could be reversibly blocked by application of 10 µM mecamylamine, a broad-spectrum antagonist of nAChRs known to inhibit ACh-induced contraction of *Ascaris* muscle [26] (Figure 3). Dose-response relationships were established for acetylcholine, nicotine and levamisole, and all responses normalised to the response to 100 µM ACh. Sigmoidal dose-response curves were fitted to the data using GraphPad Prism software (Figure 3C), and show that the order of agonist efficacy is levamisole>ACh>nicotine (Table 2), consistent with earlier voltage-clamp observations on native *A. suum* muscle tissue [8],[26]. The dose-response curves were rather shallow, with estimated Hill numbers of 0.63 for acetylcholine, 0.64 for levamisole and 0.77 for nicotine. Two explanations seemed possible; either the receptors showed negative co-operativity, which would be unusual for nicotinic receptors, or a mixed population of receptors, with differing EC_50_ for the agonists, was being produced. There is precedence for the second explanation from studies on mammalian neuronal nicotinic receptors composed of α4β2 subunits [27],[28].

**Table 2:** Summary of the properties of the receptors formed after injection of *Asu-unc-38* and *Asu-unc-29* cRNAs at different ratios.

Agonist			1∶1 *Asu-unc-38∶Asu-unc-29*	1∶5 *Asu-unc-38∶Asu-unc-29*	5∶1 *Asu-unc-38∶Asu-unc-29*	Body wall muscle contraction. Data from [11]
Acetylcholine	Not selective	*I_max_*	1.00	0.88±0.05	0.95±0.15	
		pEC_50_	4.97±0.16	5.95±0.12	6.14±0.12	ND
Levamisole	L-subtype selective	*I_max_*	1.30±0.15	1.30±0.16	0.59±0.58	
		pEC_50_	4.78±0.25	5.53±0.37	5.62±0.49	5.83±0.03
Pyrantel	L-subtype selective	*I_max_*	ND	1.03±0.27	*	
		pEC_50_	ND	6.40±0.18	*	7.24±0.03
Nicotine	N-subtype selective	*I_max_*	0.71±0.07	0.46±0.12	1.09±0.29	
		pEC_50_	4.79±0.22	5.88±0.95	5.57±1.56	4.85±0.05
Oxantel	N-subtype selective	*I_max_*	ND	*	1.44±0.88	
		pEC_50_	ND	*	5.39±1.06	7.18±0.02

The relative maximal currents (*I_max_*) have been normalized to those elicited by acetylcholine on oocytes injected with a 1∶1 ratio of the two cRNAs, due to the large variations in current size seen between individual oocytes. The acetylcholine maximal currents were up to 800 nA to 2 µA. Responses from each oocyte were normalized to 100 µM acetylcholine. ND = Not determined. Results are shown as the mean±S.E., N = minimum of 3. * Due to the small size of the responses seen, it was not possible to derive any quantitative measurements for this receptor∶agonist combination.

### Changing the subunit stoichiometry of the receptor changes its pharmacological properties

**Figure 4:**
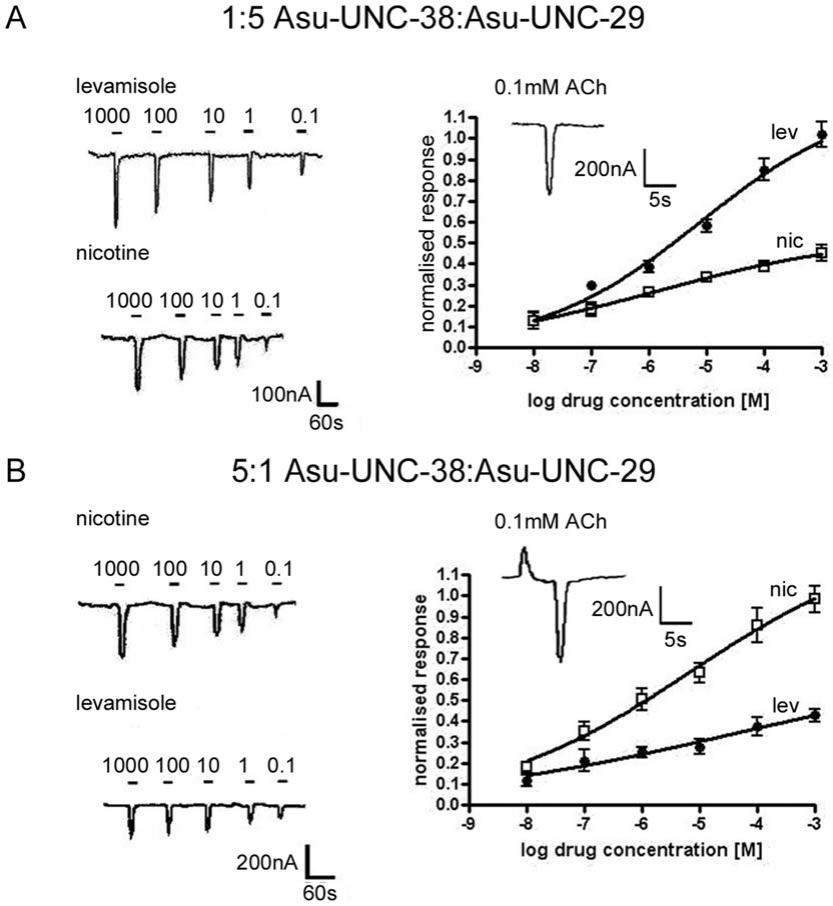
Levamisole and nicotine responses from oocytes injected with cRNAs at 5∶1 and 1∶5 ratios. A) Levamisole and nicotine responses from oocytes injected with a 1∶5 ratio of *Asu-unc-38* and *Asu-unc-29*. The currents evoked by levamisole application are larger than those produced by nicotine application. Agonist application is indicated by a bar above the trace, and the concentrations are indicated in µM. The dose-response relationships for levamisole and nicotine are derived from N = 3 n = 6 minimum for each data point, where n = number of oocytes tested, N = number of frogs from which the oocytes were obtained. Levamisole acts as a full agonist on this receptor subtype, whereas nicotine acts as a partial agonist. All responses were normalized to the response to 100 µM acetylcholine: an example of such a response is shown as the inset. B) Nicotine and levamisole responses from oocytes injected with a 5∶1 *Asu-unc-38∶Asu- unc-29* ratio. The currents evoked by nicotine application are larger than those produced by levamisole application. Agonist application is indicated by a bar above the trace, concentrations are given in µM. The dose-response relationships for levamisole and nicotine, where N = 3 n = 6 minimum for each data point. Nicotine acts as a full agonist on this receptor subtype, whereas levamisole acts as a partial agonist. All responses were normalized to the response to 100 µM acetylcholine, an example of which is shown.

The pentameric nature of the nAChR implies that when two cRNAs encoding different subunits are injected into oocytes at an equimolar ratio, a mixed population of receptors is likely to be produced (potentially (Asu-UNC-38)_5_, (Asu-UNC-38)_4_∶(Asu-UNC-29)_1_, (Asu-UNC-38)_3_∶(Asu-UNC-29)_2_, (Asu-UNC-38)_2_∶(AsuUNC-29)_3_, (UNC-38)_1_∶(UNC-29)_4_ , (UNC-38)_0_∶(UNC-29)_5_). We have already shown that molecules containing the subunits in the ratios 5∶0 and 0∶5 Asu-UNC-38∶Asu-UNC-29 do not form functional receptors (Figure 3A). The shallow slope of the dose-response relationships recorded in Figure 3C is also suggestive of a mixed population of receptors. In order to resolve this, we varied the ratio at which the *Asu-unc-38* and *Asu-unc-29* cRNAs were injected into the oocytes. Based on data from the mammalian neuronal nAChRs, this should cause the formation of a population of receptors with predominantly (UNC38)_3_∶(UNC29)_2_, (UNC38)_2_∶(UNC29)_3_ stoichiometries. Oocytes injected with the two cRNAs at ratios of 10∶1 and 1∶10 produced only very small currents in response to acetylcholine, but injection at ratios of 5∶1 and 1∶5 resulted in robust currents in response to agonist. Injection of the cRNAs in both ratios (5∶1 and 1∶5) produced receptors with comparable responses to ACh, but a striking difference in the agonist efficacy of levamisole and nicotine was observed between the two ratios (Table 2). Figure 4 shows the dose-response relationships for levamisole and nicotine (normalised to the response to 100 µM ACh) for the 5∶1 and 1∶5 (*Asu-unc38*∶*Asu-unc-29*) cRNA ratio experiments. When the ratio was 5∶1 *Asu-unc-38*∶*Asu-unc-29* nicotine was a full agonist, whereas levamisole was only a partial agonist, with a maximal response of only 60% of that of ACh (Table 2). When cRNAs at a ratio of 1∶5 *Asu-unc-38*∶*Asu-unc-29* were used, levamisole was now a full agonist (with a maximal response of 130% of that of ACh), and nicotine was now only a partial agonist with a maximal response of only 45% (Table 2).

**Figure 5:**
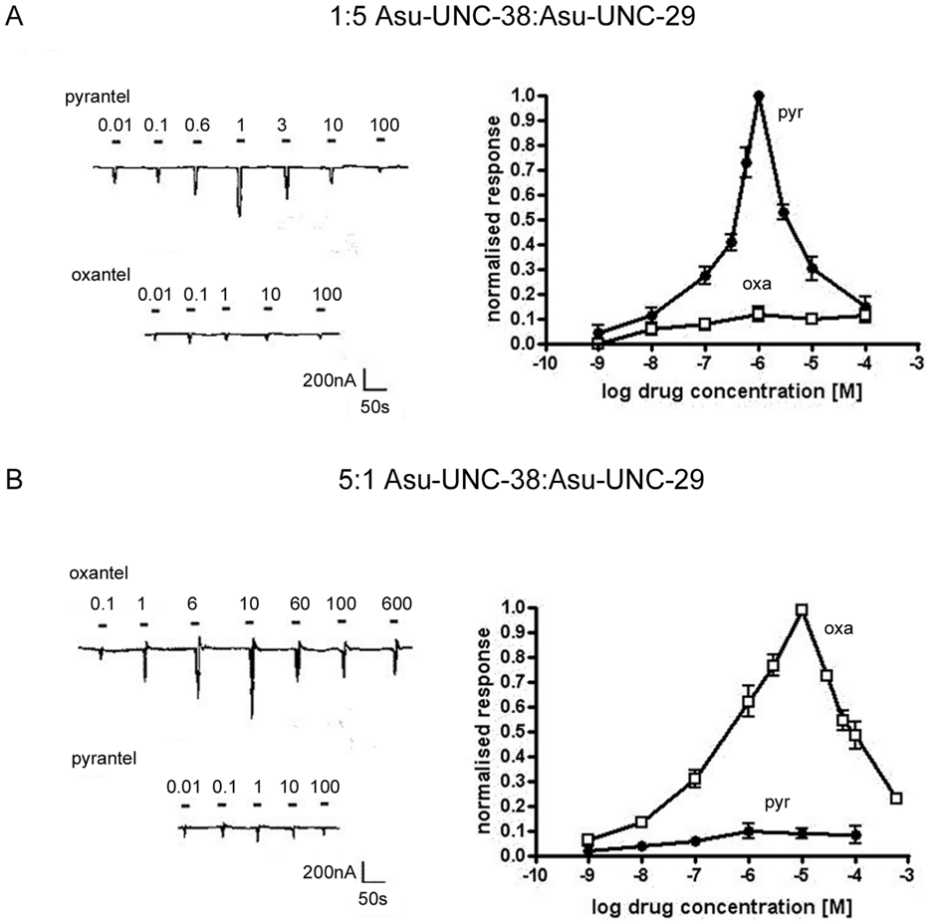
Responses to the anthelmintics pyrantel and oxantel. A) Electrophysiological recording from an oocyte injected with a 1∶5 *Asu-unc-38∶Asu-unc-29* ratio in response to the application of pyrantel (upper trace) and oxantel (lower trace). Pyrantel clearly acts as an agonist at lower concentrations (<1 µM) and a channel block effect reduces the response seen at higher concentrations; in comparison to this oxantel only produced small currents at any concentration. Agonist application is indicated by a bar above the trace and the concentrations are given in µM. The dose-response relationships for pyrantel and oxantel from oocytes injected with a 1∶5 ratio of *Asu-unc-38* and *Asu-unc-29* are shown: N = 3 n = 6 minimum for each data point. All responses were normalized to the maximal response (i.e. to the response to 1 µM pyrantel), which was comparable to the response to 100 µM acetylcholine (not shown). B) Electrophysiological recording from oocytes injected with a 5∶1 *Asu-unc-38∶Asu-_unc-29* ratio in response to the application of oxantel (upper trace) and pyrantel (lower trace). Oxantel clearly acts as an agonist at lower concentrations (<10 µM) and a channel block effect reduces the response seen at higher concentrations; in comparison to this pyrantel only produced small currents at any concentration. Agonist application is indicated by a bar above the trace. Agonist concentrations and the concentrations are given in µM. The Dose-response relationships for pyrantel and oxantel from oocytes injected with a 5∶1 ratio of *Asu-unc-38* and *Asu-unc-29* are based on N = 3 n = 6 minimum for each data point. All responses were normalized to the maximal response (i.e. to the response to 10 µM oxantel), which was comparable to the response to 100 µM acetylcholine (not shown).

Additional experiments were performed to further characterise the pharmacology of the different receptors produced using 5∶1 and 1∶5 ratios of *Asu-unc-38*∶*Asu-unc-29* cRNAs. Pyrantel and oxantel, in addition to their respective specificity for the L- and N- subtypes native nAChR found in *A. suum* [11], are anthelmintic compounds of medical and veterinary importance. The dose-response relationships observed using these compounds were more complex than observed for the other agonists as both oxantel and pyrantel are known to be agonists of native *A. suum* nAChRs at lower concentrations, but open channel blockers at higher concentrations, producing an asymmetric bell-shaped dose-response relationship [8],[29]. The results for these experiments are shown in Figure 5, and show dramatic differences in agonist efficacy between the receptors with different stoichiometries. The oocytes injected with a 5∶1 ratio of *Asu-unc-38*∶*Asu-unc-29* cRNAs responded to oxantel, giving a maximal response at ∼10 µM, but gave very little response to pyrantel. The oocytes injected with a 1∶5 ratio of *Asu-unc-38*∶*Asu-unc-29* cRNAs were more sensitive to pyrantel, giving a maximal response at ∼1 µM, but gave a negligible response to oxantel. Maximal responses in both cases were comparable to the maximal response to ACh (Table 2), though the steep nature of the dose-response curves suggests that full agonist activity was not reached before the channel blocking action began to affect response size. The specificity of levamisole and pyrantel for the receptor population formed from a 1∶5 *Asu-unc-38*∶*Asu-unc-29* cRNA ratio matches the pharmacological properties of the native *A. suum* L-subtype nAChR, whereas the specificity of nicotine and oxantel for the receptor population formed following injection of a 5∶1 cRNA ratio resembles the pharmacology of the N-subtype nAChR.

## Discussion

The generation of functional levamisole-sensitive nAChRs using the *A. suum* orthologues of UNC-38 and UNC-29 shows that, as in *C. elegans*, these subunits are likely to be components of the native levamisole-sensitive nAChR [15]. However there are clear and striking differences between the two species in terms of the number of gene products that need to be expressed in order to reconstitute a robust levamisole response. In *C. elegans*, 3 other nAChR subunits and three ancillary proteins are necessary for the full levamisole response: LEV-1, LEV-8, UNC-63, RIC-3, UNC-50 and UNC-74 [17]. We have previously shown using a bioinformatic approach and the genome of *B. malayi*, a parasite closely related to *A. suum*, that *lev-1* and *lev-8* are apparently absent from this group of nematodes; however a sequence related to *unc-63* is apparently present in *B. malayi* [21], though its function is currently unknown. In addition, Asu-UNC-29 and Asu-UNC-38 form nicotine-sensitive receptors. In *C. elegans* nicotine sensitivity is conferred by ACR-16 subunit-containing receptors [18], and the levamisole-sensitive receptor is completely insensitive to nicotine [17]. We have so far been unable to identify any sequences clearly orthologous to *acr-16* in *A. suum* or *B. malayi* [21], though it is possible that other nAChR subunits may fulfil an analogous function in the parasites: in *C. elegans acr-16* belongs to a fairly large sub-family of nAChR subunit genes [30], and the other parasite genomes seem to possess at least one member of this sub-family [21]. These results demonstrate very clear and marked differences between the nAChR of *C. elegans* and an important parasite and emphasise the importance of studying anthelmintic receptors in target species in addition to model organisms. They also raise the possibility that parasite-specific screens for novel cholinergic anthelmintics could be developed via the expression of the parasite receptor subunits.

The two subunit sequences possessed all of the features we would expect from functional equivalents of *C. elegans* UNC-38 and UNC-29, including the amino-acids thought to confer sensitivity to low concentrations of levamisole [24]. Immunoflourescence labelling of isolated *A. suum* muscle cells and nerve cords supported the proposal that the two subunits are expressed on muscle cells, including the neuromuscular junction. Nicotinic receptors in *A. suum* muscle, unlike those in *C. elegans*, are not clustered solely at the neuromuscular junction, but are present all over the cell membrane, as shown both in Figure 2 and by the fact that it is possible to make single-channel and intracellular recordings from the bag region of the cell [10]–[12],[31].

**Figure 6:**
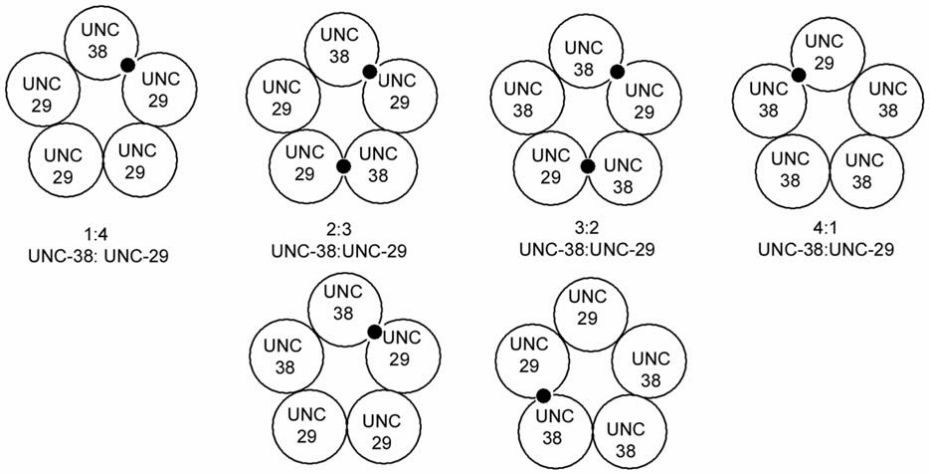
Possible combinations of the two subunits in the oocyte membrane. The possible combination of Asu-UNC-38 and Asu-UNC-29 that might be present in the injected oocytes are shown. Agonist binding sites, which, by analogy with mammalian receptors should be at an interface between the two subunits, [38] are indicated by black circles. The Figure is based on a similar discussion for mammalian receptors [32].

We could express cRNAs encoding the two *A. suum* subunits in *Xenopus* oocytes to form receptors with many of the properties reported for the muscle nicotinic receptors; they were sensitive to aceylcholine, levamisole and nicotine. However, injection of the two subunit cRNAs into the oocytes at different ratios generated distinct receptor populations. This effect was more obvious when ratios of 1∶5 and 5∶1 (*Asu-unc-38∶Asu-unc-29*) were injected; injection of the two cRNAs at ratios of 1∶10 and 10∶1 produced very small currents which were impossible to study further. Though several different populations could theoretically be generated in these experiments [32] (Figure 6), previous data from the mammalian α4β2 nAChR suggests that the most likely combinations of two subunits to yield functional receptors will be in the ratios 2∶3 and 3∶2 [27],[28],[33] and this is supported by our difficulty in producing robust responses when injecting at a ratio of 10∶1, which would theoretically produce more receptors of a 4∶1 than 3∶2 subunit stoichiometry. If the pharmacology of *A. suum* nAChRs can be altered simply by generating different combinations of two subunits, this may help to explain how sufficient pharmacological and neurological complexity can be found in parasites with remarkably few nAChR genes compared to *C. elegans*. Pharmacological diversity generated by alternate stoichiometries has not previously been observed in invertebrates, but is well described for heterologous expression of the mammalian neuronal nAChR subunits α4 and β2. Receptors with the stoichiometry (α4)_3_(β2)_2_ have lower agonist affinity and a different pharmacological profile to receptors with the stoichiometry (α4)_2_(β2)_3_, and addition of an accessory subunit to give (for example) an (α4)_2_(β2)_2_(α5) combination confers additional pharmacological differences [27],[28],[33]. In extrapolating the mammalian α4β2 model to interpret the results presented here, we suggest that we have generated receptor subtypes that have the stoichiometry (Asu-UNC-38)_2_(Asu-UNC-29)_3_, and (Asu-UNC-38)_3_(Asu-UNC-29)_2_.

If our results are compared with those obtained from single channel recordings on *A. suum* muscle [11]–[13], our data are consistent with a suggestion that (Asu-UNC-38)_2_(Asu-UNC-29)_3_ constitutes the L-subtype nAChR and that (Asu-UNC-38)_3_(Asu-UNC-29)_2_ forms the N-subtype. The complex mixture of receptors present in the *in vivo* preparation makes a quantitative comparison with our *in vitro* data difficult, but there are clear qualitative similarities. The L-subtype of the native receptor is preferentially activated by levamisole, though nicotine does also open L-type channels, and *vice versa* for the N-subtype; our oocyte data show that nicotine is only a partial agonist at the putative (Asu-UNC-38)_2_(Asu-UNC-29)_3_ receptor and levamisole is, similarly, only a partial agonist at the putative (Asu-UNC-38)_3_(Asu-UNC-29)_2_ receptor. There are also clear similarities in the effects of the anthelmintics, pyrantel and oxantel, between our results and the single channel measurements; pyrantel acts at the same sub-type that levamisole prefers and oxantel at the one preferred by nicotine. We believe, therefore, that we have recapitulated many of the properties of the L- and N-subtypes by varying the expression levels of the two *A. suum* subunits within the oocyte system. These results have important implications for the development and management of parasite resistance to cholinergic anthelmintics; it has previously been assumed that parasites resistant to levamisole or pyrantel may still be susceptible to oxantel as different receptor sub-types are involved [11], but if both receptor types contain the same subunits, then the potential for development of resistance to both classes of cholinergic anthelmintic is greater. Kopp et al [34] have recently reported changes in the expression of hookworm nicotinic receptor subunit mRNAs that may be associated with pyrantel resistance.

An important open question concerns the function of the *A. suum* UNC-63 subunit. Despite exhaustive efforts, so far we have been unable to generate a cDNA clone encoding an obviously full-length UNC-63 subunit. This may be due to purely technical difficulties, or it may that, in *A. suum*, *unc-63* encodes only a truncated mRNA. It is tempting to speculate that the B-subtype nAChR has the composition (Asu-UNC-38)_2_(Asu-UNC-29)_2_(Asu-UNC-63)_1_, by analogy with the (α4)_2_(β2)_2_(α5)_1_ receptors found in mammals [33], but we have no direct evidence to support this speculation.

In summary, we have demonstrated that the *A. suum* homologues of UNC-38 and UNC-29 co-express in *Xenopus* oocytes to form a functional nAChR, which is the first successful expression *in vitro* of a nAChR from parasitic nematodes. This will create opportunities for future work to be performed, where for the first time candidate drug resistance mutations in parasite nAChR subunits can be assessed directly for their effect on anthelmintic efficacy and receptor function. This work also paves the way to an accelerated understanding of anthelmintic drug targets and the potential development of parasite-specific target-based screens for new compounds.

## Materials and Methods

### Ethics statement

All animals used for production of antisera were handled in strict accordance with good animal practice and the conditions defined by the United Kingdom Home Office (Harlan, UK) or the United States Department of Agriculture (Sigma Genosys).

### Molecular biology

Partial sequences of *Ascaris suum unc-38* and *unc-29* were amplified using primers designed on a partial sequence of *Asu-unc-38* present in GenBank (AJ011382) (Primer sequences GTCGCGCTTACCGTTTTCTTCC and CCATCGCCACATATTTCCAGTCTT) and using degenerate primers based on an alignment of the *unc-29* sequences from *C. elegans* and *Brugia malayi* (primer sequences ATCAAYGTNGAYGARAARGAYCA and ATYTCRTTYTCRTTRTANGTCCA). The sequences were extended to full length using 5′ and 3′ RACE [22]. For 5′ RACE, an oligonucleotide corresponding to the spliced leader, SL1 (GGTTTAATTACCCAAGTTTGAG) was used in conjunction with specific internal primers (*Asu-unc38* TAAAGCACGCTGACACCACC; *Asu-unc-29* ATYTCRTTYTCRTTRTANGTCCA). For 3′ RACE an ‘anchor’ primer sequence incorporated into the oligo(dT) primer used for reverse transcription (GACCACGCGTATCGATGTCGAC) was together with an internal primer specific for *Asu-unc-29* (GCACTAAGAGCTATTGACGCG)). Full length cDNAs were amplified using specific primers (*Asu-unc-38* CTGCATTTATTAAGATGTTTGG and ATGTAAATTATTGAGTGACTGG; *Asu-unc-29* CACTGAGGGCAGTTATGCACC and CAGTGTGGGCGAGATATTAGATC). Full length cDNAs were cloned into pGEM-T (Promega) and transformed into XL-1 blue competent cells (Stratagene).

### Immunofluorescence

Antisera were raised, in rabbit and goat respectively, against subunit-specific N-terminal peptides from Asu-UNC-38 and Asu-UNC-29 (Harlan, UK and Sigma-Genosys, USA) then antibodies were affinity purified as described [35]. Adult *A. suum* collected from a local abattoir were kindly supplied by Professor Aaron Maule and Emma Kidd from Queen's University, Belfast. Muscle cells were prepared by collagenase digestion [36] fixed and antibody labelled [37]. Muscle cell preparations were then mounted on slides and viewed using a Zeiss 510 confocal laser scanning microscope.

### Oocyte expression and electrophysiology

*A. suum unc-38* and *unc-29* cDNAs were sub-cloned into the expression vector pT7TS (obtained from P. Krieg, University of Texas, Addgene plasmid 17091), containing 5′ and 3′ untranslated regions from *Xenopus* β-globin. Plasmids were linearised, and then used as template for cRNA synthesis using the T7 mMessage mMachine kit (Ambion, UK). Oocytes were obtained from EcoCyte Biosciences, Germany. Oocytes were injected with nicotinic receptor cRNAs in RNAse-free water in a total volume of 50 nl, which was the same for all experiments. When a single cRNA species was injected, 25 ng was used; when both *Asu-unc-29* and *Asu-unc-38* cRNAs were injected, a total of 50 ng was used. Oocyte injection was carried out using a Drummond ‘Nanoject’ microinjector and electrophysiological experiments were carried out [38] using a GeneClamp 500 amplifier and Digidata 1322A (Axon Instruments) with oocytes being voltage-clamped at −60 mV. Pharmacological compounds were obtained from Sigma. Data was recorded and responses measured using pClamp software. GraphPad Prism software was used to analyse the data and fit sigmoid dose-response curves to the equation *I/I_max_* = (*I_max_*−*I_min_*/[1+10^(logEC50−[ag]*nH)^])×*I_max_*. For pyrantel and oxantel, the equation was fitted to the data obtained for the rising phase of the response only.

### Accession numbers

The Accession numbers for proteins mentioned in the text are: *C. elegans*: UNC-38 – Q23022, UNC-*29* –P48181, UNC-63 – Q9N587, ACR-12 – Q9GQU9, ACR-8 – Q23355, ACR-6 – Q9N4M3, LEV-1 – P48181, ACR-3 – Q93149, ACR-2 – P48182, ACR-16 – P48180. *B. malayi*: BM_UNC-29 – Bm1_35890.
